# Cone‐beam computed tomography evaluation of C-shaped root and canal morphology of mandibular premolars

**DOI:** 10.1186/s12903-021-01596-y

**Published:** 2021-05-04

**Authors:** Gisbeli Brea, Francisco Gomez, Jose Francisco Gomez-Sosa

**Affiliations:** Postgraduate Department of Endodontics, Universidad Central de Venezuela, Caracas, Venezuela

**Keywords:** Mandibular first premolars, Mandibular second premolars, Anatomical variation, C-shaped configuration, CBCT

## Abstract

**Background:**

Mandibular premolars are complicated teeth to endodontically treat due to the anatomical variations that can present. The purpose of this study was to determine the presence of C-shaped configurations in mandibular premolars by cone-beam computed tomography (CBCT).

**Methods:**

380 mandibular first premolars and 308 mandibular second premolars cone-beam computed tomographic images were obtained from 292 patients (175 female and 117 male). Tooth position, number of roots, root canals, C-shaped root canal system configuration, level of canal bifurcation and radicular grooves were evaluated by two endodontists trained in CBCT evaluation; previously calibrated between them, and a radiologist with endodontic experience. Data were statistically compared by The Chi-square test (α = 0.05) to examine any significant difference between gender and C-shaped root canal system and any significant difference between C-shaped configuration according to Fan criteria and gender.

**Results:**

Overall 100% (n = 688) teeth examined, 19.2% (n = 132) had a C-shaped root canals system. 9.16% (n = 63) in male patients and 10.03% (n = 69) in female patients. The prevalence of C-shaped root canal system in mandibular first premolar was 83.33 and 16.66% in mandibular second premolars. According to Fan classification, the mandibular first premolars showed 3.63% as C1, 9.09% C2, 59.09% C3, 21.81% C4a, 1.8% C4b. Mandibular second premolars showed 13.63% as C1, 18.18% C2, 13.63% C3, 27.27% C4a, 9.09% C4b. Male patients showed 4.54% as C1, 3.78% C2, 22.72% C3, 11.36% C4a, 0.75% C4b, and 4.54% out of classification. Female patients showed 0.75% as C1, 6.81% C2, 30.03% C3, 9.84 C4a, 2.27% C4b, and 2.27% out of classification. The 53.36% canal bifurcation in mandibular first premolars and 50.09% in mandibular second premolars were in the middle third. No statistical differences were found between C-shaped root canal system and gender or C-shaped configuration according to Fan criteria and gender. P was < 0.05.

**Conclusions:**

Within the limitation of this study, the presence of C-shaped anatomical system is relatively low in mandibular second premolars than in mandibular first premolars.

## Background

Mandibular premolars are complicated teeth to endodontically treat due to the anatomical variations that can present. Knowledge the morphology of the mandibular premolars along with a correct diagnosis is a prerequisite for successful treatment in endodontic therapy [1, 2]. The cross-section of the pulp chamber in the mandibular premolars is oval, and the root canal system is broader in the buccal-lingual sense than the mesio-distal direction. They have two pulpal horns, one large pointed buccal and one small rounded lingual for the first premolar and more prominent for the second [3, 4].

Some studies have reported anatomical variations of this dental group, such as the presence of two or more roots, more than one canal and the presence of bifurcations of the root canal system [5–11]. Other studies have reported unusual anatomy in the mandibular premolars described as radicular grooves that cause a C-shaped morphology when these teeth are analyzed in their cross-sections [1, 3, 4, 8, 9, 11, 13–27]. This anatomy not always is continuous from the entrance of the canal to the apical foramen. The main anatomical characteristic is the presence of isthmuses that connect the individual canal, and that can vary along their root [22]. C-shaped root morphology present narrow canals, roots concavities, and dentinal thinness walls so their cleaning and conformation required a carefully treatment planning and clinical knowledge to avoid procedure mistakes [13].

It has been hypothesized that C-shaped root canal anatomy is due to a reduced dentine speed formation on the lingual side, it is also thought to be a failure of Hertwig’s epithelial root sheath to fuse during the stage of tooth development or coalesce by the continuous cementum deposition [6].

Cooke and Cox first used the C-shaped term in 1979, and reclassifications that are more detailed have emerged for mandibular premolars, such as Fan et al. also based on the shape of the root cross-section [23].

For the analysis of premolars with C-shaped configuration, different study techniques of their characteristics have been implemented; including clarification, cross-sections and microcomputed tomography in teeth that have been extracted [1, 2, 17].

In vivo analysis techniques can be performed with conventional periapical radiographs, which lack detail by providing a two-dimensional image an insufficient data for the analysis of this cases. The best method for an accurate determination of their roots morphology is the CBCT. The images provided by CBCT have a determining effect on the identification anatomical variations, which allows the endodontist or clinician to make better diagnoses and decision-making prior to the start of treatment [28–30]. This accurate tool offers three planes of analysis of the teeth that show the variations, which are characterized due to the presence of isthmus, invaginations and bifurcations that are impossible to detail with a conventional image [1, 2, 17].

CBCT evaluations have made it possible to carry out studies of mandibular premolars in populations such as China, India, Iran, USA and Finland, allowing them to know the characteristics of anatomical variations, and the percentage of appearance of the population studied. The present study aimed to determine the presence of C-shaped configurations (root and root canal) in mandibular premolars by CBCT in a Venezuelan population.

## Methods

### Sample selection

The Ethics Committee of Dentistry School to the Universidad Central de Venezuela approved this study. The CBCT images of mandibular premolars were acquired from patients who required a preoperative assessment as part of their dental examination, diagnosis, and treatment planning from imaging diagnostic center in Caracas, Venezuela between January 2014 and December 2017.

A total of 380 mandibular first premolars and 308 mandibular second premolars from 292 patients were select based on the following criteria: presence of CBCT images of mandibular premolars with complete root formation, presence of high-quality CBCT images, absence of root canal treatment and absence of root resorption or periapical lesions.

### Image acquisition

The CBCT images were obtained using Kodak 9000 3D unit (Carestream Dental, Atlanta, GA, USA); at 60–90 kV and 2–15 mA with an exposure time of 2–6 s. The voxel size of the images was 76 × 76 × 76; and the slice thickness was 200 μm with 16 bits grayscale. An experienced radiologist performed the acquisition process according to the manufacturer’s recommended protocol with the minimum exposure necessary for adequate image quality. According to human ethics procedures, all methods were carried out in accordance with relevant guidelines and regulations.

#### Image evaluation

All the images from 688 mandibular premolars were evaluated with a 3D Imaging Software 3.3.9.0 (Carestream Dental LLC, Atlanta, GA. USA) and a Dell Inspiron 15 5000 Laptop (Intel® Core™ i5-1135G7, Processor 8 MB Cache, up to 4.2 GHz. Windows 10 Pro 64-bit English).

Two endodontists independently evaluated the images twice, with a week interval between the assessments. If there were disagreements between them, a radiologist with endodontic experience was asked to perform a third evaluation and then reach a final consensus. All the evaluators were calibrated by analyzing 20 random cases of mandibular premolars based on the same criteria and variants. The Cohen´s Kappa was used to analyzed Presence of anatomical variation and variation type, and the intraclass correlation coefficient (ICC) was used to analyzed the roots and root canals number.

Results of the first analysis showed high values of agreement with the statistical methods applied: Presence of Anatomical Variations: 0.91 Cohen’s Kappa, Variation Type: 0.92 Cohen’s Kappa, Roots Number: 0.89 ICC and root canals number: 0.91 ICC. Results of second calibration between specialists showed same high values ​​ of agreement: Presence of Anatomical Variations: 0.90 Cohen’s Kappa, Variation Type: 0.94 Cohen’s Kappa, Roots number: 0.91 ICC and root canals number: 0.89 ICC.

Then the following information of 688 mandibular premolars were recorded:

- Tooth position: first or second mandibular premolar.
- Number of roots and root canals.
- C-shaped root canal system configuration.
- C-shaped classification according to Fan et al. [22] criteria:
  - C1: the shape was a continuous “C” with no separation or division.
  - C2: the canal shape resembled a semicolon resulting from a discontinuation in the “C” outline.
  - C3: two separate round, oval, or flat canals.
  - C4: only one round, oval, or flat canal in that cross-section, which was further classified into three subdivisions:
  - C4a (round canal): the long canal diameter almost equal to the short diameter.
  - C4b (oval canal): the long canal diameter was at least 2 times shorter than the short diameter.
  - C4c (flat canal): the long canal diameter was at least two times longer than the short diameter.
  - C5: three or more separate canals in the cross-section.
  - C6: no canal lumen or no intact canal could be observed.
- Level of canal bifurcation: cervical, middle or apical third.
- Radicular grooves: mesial, distal, buccal or lingual area.

The Chi-square test ((α = 0.05) was used to examine any significant difference between gender and C-shaped root canal system configuration and any significant difference between C-shaped configuration according to Fan et al. [22] criteria and gender by SPSS 21.0 software (SPSS Inc, Chicago, IL) Differences were statistically significant when P was < 0.05.

## Results

### Mandibular first premolars general features

Three hundred eighty mandibular first premolars were evaluated. Their root and root canals configurations were analyzed, and most of them showed a single root in 91.84% of the samples, two roots in 7.63%, and only 0.52% three roots. The canals number in the sample studied were 68.7% a single canal, 30.5% two canals, and 0.8% three canals.

### C-Shaped configuration mandibular first premolars

**Fig. 1:**
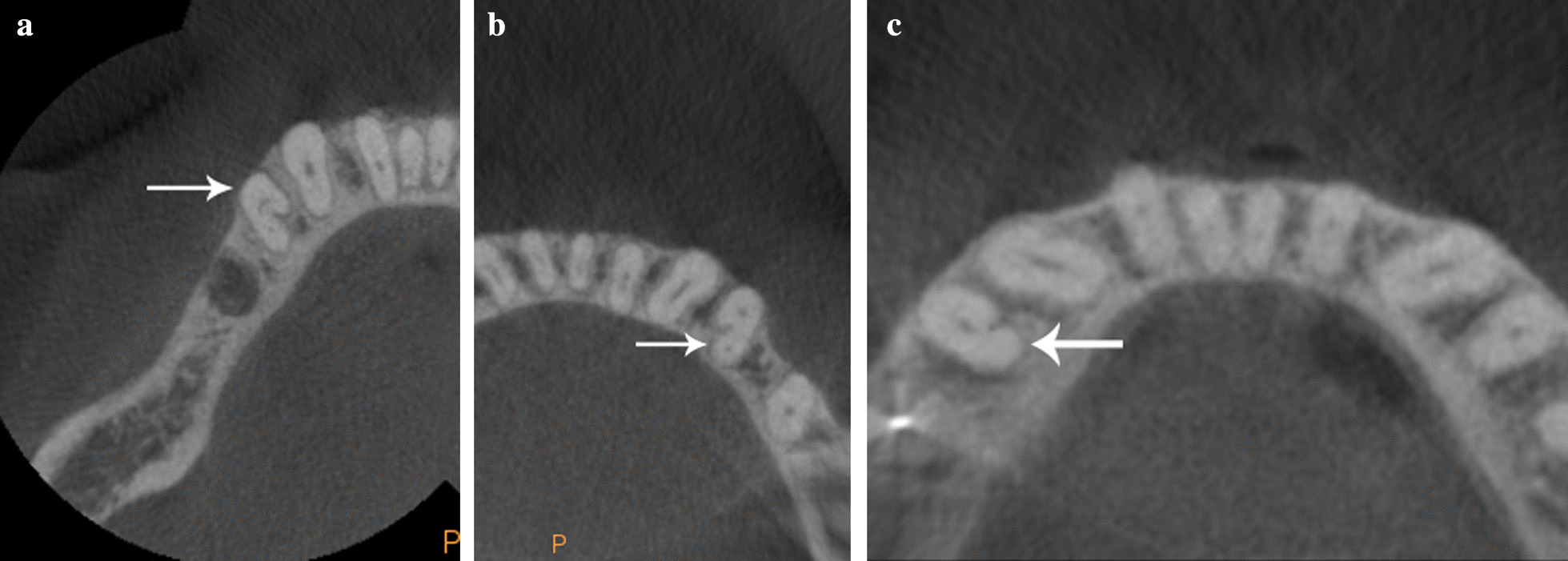
Axial views of examples of the three most frequent classifications found in this study in C-shaped mandibular first premolars: **a** C2, **b** C3 and **c **C4a

**Fig. 2:**
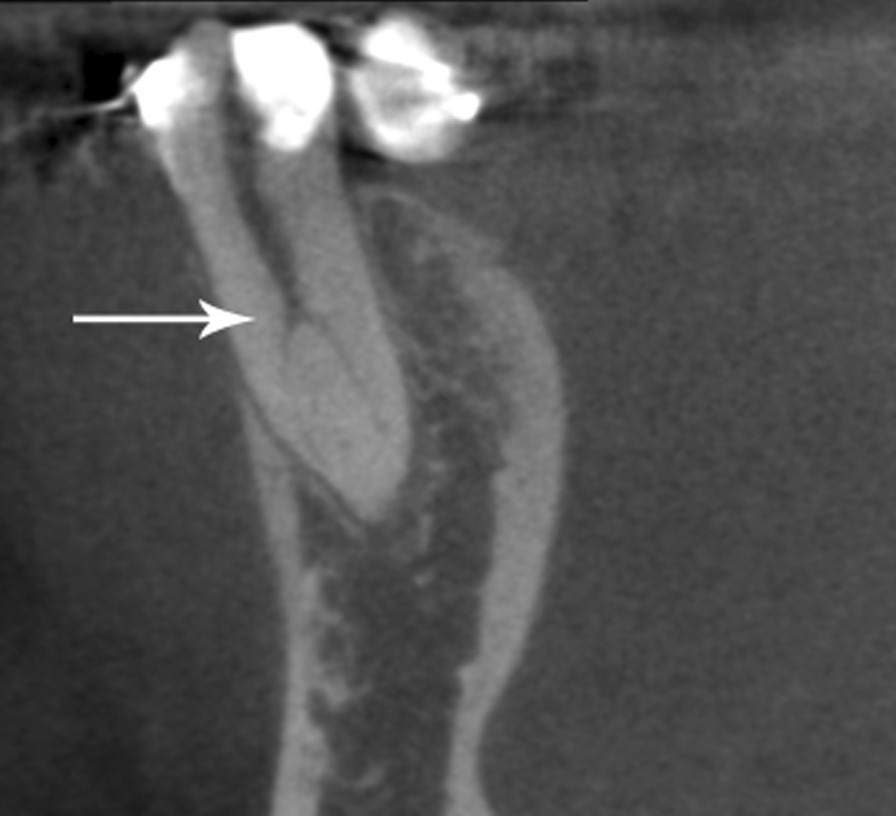
Sagittal view of the C-shaped mandibular first premolar representative of the furcation area most found in this study, located in the middle third of the root

Presence of C-shaped root configurations was observed in 110 mandibular first premolars representing 28.94% of the samples. According to Fan et al. [22] classification these C-shaped premolars were identified as C1 3.63%, C2 9.09% (Fig. 1a), C3 59.09% (Fig. 1b), C4a 21.81% (Fig. 1c), C4b 1.8%, and 4.54% as unclassified variation. The root canals number in these samples showed 10% with a single canal, 87.27% two canals and 2.72% three canals. Level of canals bifurcation showed 25% bifurcated in the cervical third, 56.36% middle third (Fig. 2), 8.18% apical third, and 10% were not present. Detailed canals bifurcation is presented according to Fan et al. classification [22] (Table 1). Radicular grooves in the C-shaped premolars under study were found as follow: 84.5% in mesial area, 0.9% buccal area, 11.8% lingual area, and 2.7% simultaneous grooves in buccal and lingual area.

**Table 1:** C-shaped mandibular first premolars according to Fan et al. classification, and canals bifurcation

C-shape classification			Canals bifurcation		
	Total (%)	Cervical third	Middle third	Apical Third	Without bifurcation
Total	110 (100%)	27 (25%)	62 (56.36%)	9 (8.18%)	11 (10%)
C1	4 (3.63)	0	3 (2.72%)	1 (0.90%)	0
C2	10 (9.09)	2 (1.81%)	7 (6.36%)	2 (1.81%)	0
C3	65 (59.09)	21 (19.09%)	43 (39.09%)	1 (0.90%)	0
C4a	24 (21.81)	3 (2.72%)	5 (4.54%)	4 (3.63%)	10 (9.09%)
C4b	2 (1.8)	0	1 (0.90%)	0	1 (0.90%)
Could not be classified	5 (4.54)	1 (0.90%)	3 (2.72%)	1 (0.90%)	0

### Mandibular Second premolars general features

Three hundred eight mandibular second premolars were evaluated. Their root and root canals configurations were analyzed and most of them showed a single root in 98.05% of the samples, two roots in the 1.62%, and only 0.32% three roots. The canals number in the sample studied were 92.86% a single canal, 6.50% two canals, and 0.64% three canals.

### C-Shaped configuration mandibular second premolars

**Fig. 3:**
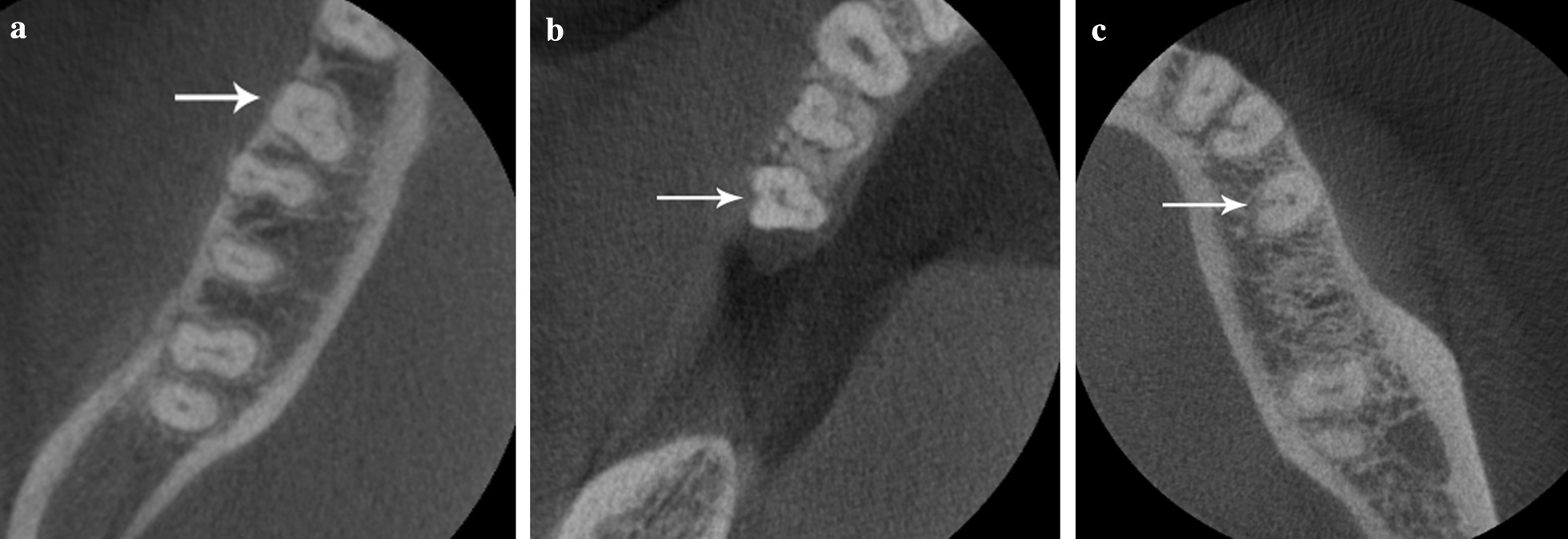
Axial views of examples of the three most frequent classifications found in this study in C-shaped mandibular second premolars: **a**  C1, **b** C2 and **c** C4a

**Fig. 4:**
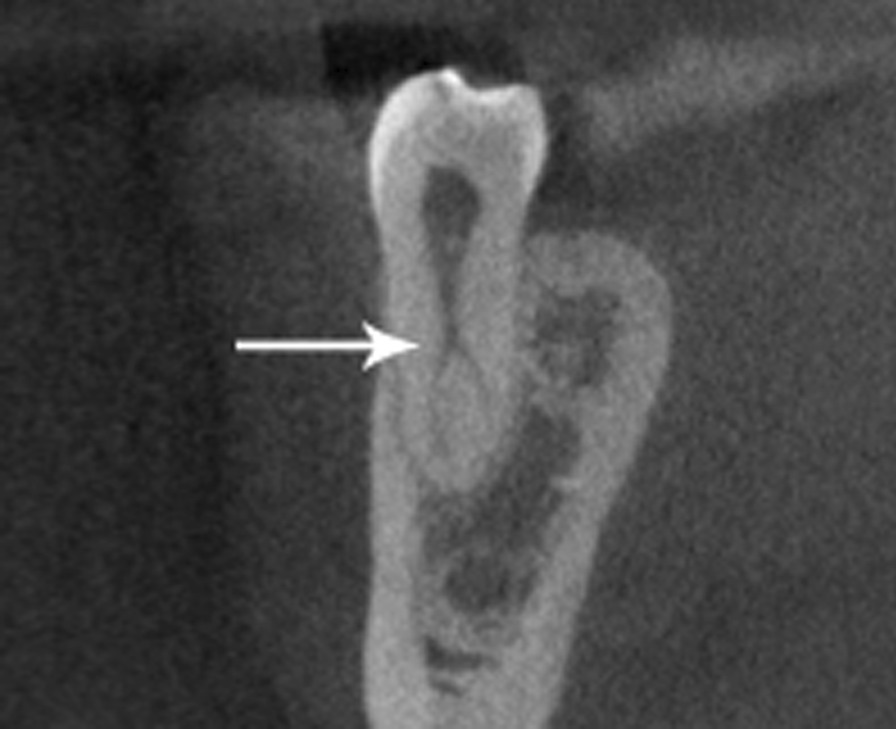
Sagittal view of the C-shaped mandibular second premolar representative of the furcation area most found in this study, located in the middle third of the root

Presence of C-shaped root canal system was observed in 22 mandibular second premolars in 7.14% of the samples. According to Fan et al. [22] classification these C-shaped premolars were identified as C1 13.63% (Fig. 3a), C2 18.18% (Fig. 3b), C3 13.63%, C4a 27.27% (Fig. 3c), C4b 9.09% and 18.18% as unclassified variation. The root canals number in these samples showed 22.72% with a single canal, 68.18% two canals, and 9.09% three canals. Level of canal bifurcation showed 13.63% bifurcated in the cervical third, 59.09% the middle third (Fig. 4), 4.54% apical third, and 22.72% were not present. Detailed canal bifurcation is presented according to Fan et al. classification [22] (Table 2). Radicular grooves in the C-shaped premolars under study were found as follow: 50% in the mesial area, 14% in buccal area, and 36% in the lingual area.

**Table 2:** C-shaped mandibular second premolars according to Fan et al. classification, and canals bifurcation

C-shape classification			Canals bifurcation		
	Total (%)	Cervical third	Middle third	Apical Third	Without bifurcation
Total	22 (100%)	3 (13.63%)	13 (59.09%)	1 (4.54%)	5 (22.72%)
C1	3 (13.63)	0	3 (13.63%)	0	0
C2	4 (18.18)	1 (4.54%)	3 (13.63%)	0	0
C3	3 (13.63)	1 (4.54%)	2 (9.09%)	0	0
C4a	6 (27.27)	0	2 (9.09%)	1 (4.54%)	3 (13.63%)
C4b	2 (9.09)	0	0	0	2 (9.09%)
Could not be classified	4 (18.18)	1 (4.54%)	3 (13.63%)	0	0

According to the gender and C-shaped configuration to Fan criteria to the 100% (n = 132) of the C-shaped samples studied, male patients showed 47.72% and female patients 52.27% (Table 3). No significant difference was found between C-shaped root canal system configuration and gender or C-shaped configuration according to Fan criteria and gender (P > .05).

**Table 3:** C-shaped root canal system in mandibular premolars according to Fan et al. classifications, and gender

C-shaped mandibular premolars	Male (%)	Female (%)
C1	6 (4.54%)	1 (0.75%)
C2	5 (3.78%)	9 (6.81%)
C3	30 (22.72%)	40 (30.03%)
C4a	15 (11.36%)	13 (9.84%)
C4b	1 (0.75%)	3 (2.27%)
Out of classification	6 (4.54%)	3 (2.27%)
Total (%)	63 (47.72%)	69 (52.27%)

## Discussion

It is necessary to have in-depth knowledge in mandibular premolars, their wide anatomic variations and canal morphology to apply clinical skills for its proper treatment [6, 13, 18]. The American Endodontic Association (AAE) and the American Academy of Oral and Maxillofacial Radiology (AAOMR) have suggested the use of Cone Beam Computed Tomography in endodontics to identify abnormalities or variations where complex morphology is suspected, based in conventional radiographs [31].

Conventional intraoral radiographs are routinely used to assess root canal anatomy, but in cases with principal canal fuzziness in any third, the best method for accurate determination of this root canal morphology is CBCT; 3D images provide maximum information to the clinician of the canal shape along the root and cross-sectional details of the tooth [9, 14, 15].

Venezuela is a variety ethnic country, to our knowledge it does not have statistical studies about C-shaped mandibular premolars; the only available information were cases reports with this configuration [6, 13]. For this reason, the present study used a database from patients of a Venezuelan imaging diagnostic center to analyze this anatomical variation and their characteristics.

Based on the CBCT analysis of this database, the most frequent morphology in the mandibular first premolars was a single root, in more than 91% of the cases and 67% a single canal; similar to other studies [3, 4, 10, 11, 18, 19, 32]. Two roots in 8% of the samples, higher than Cleghorn, Rahimi and Yu et al. studies [3, 4, 19], and lower than Bürklein, Zillich, Tian and Huang et al. [2, 10, 11, 32]. 0.52% showed three roots, which matches with other studies [3, 10, 11, 32]. 30.5% samples had two canals, it was in discrepancy with other studies whose appearance rate was around 20% [3, 4, 10, 18, 32]. Only 0.8% showed three canals, in approximate ranges with the Zillich and Yu et al. studies [10, 19]. The C-shaped root canal system was represented 29%, similar with other Chinese population studies: 24%, 27.8% [22, 31] And different with 14% in the USA [20], 0.55%, 1.14%, and 4.1% in China [18, 19, 26], 0.92%, and 10% in India [17, 25], 1.4%, and 2.4% in the Iranian population [4, 27], as well as 9% in the Finnish population [16]. This discrepancy may be due to differences in races, the number of samples, analysis technique, and application of statistical parameters. The highest C-shape root canal system according to Fan criteria [22] was C3, similar to Khedmat et al. study [27]. The most frequent canal bifurcation was in the middle third, similar with Liu and Jaju et al. results [9, 31]. Radicular grooves were located in the mesial area nearly to the lingual zone, similar to other findings [1, 9, 20–22, 25, 27].

This study reported five cases of mandibular first premolars that could not be classified according to Fan criteria. Three with a single root and two canals; two with three roots and three canals. Canal bifurcation was located in three cases in the middle third, one in cervical third and other in apical third. Radicular grooves were located in the mesial and lingual area in two sample, one case showed buccal and lingual area simultaneously.

Based on the CBCT analysis of this database, the most frequent morphology in the mandibular second premolars was a single root in more than 91% of the cases and 92% a single canal, similar to other studies [10, 11, 19]. Two roots in 1.62% of the samples, higher than Cleghorn study [11] and lower than Bürklein study [2]. 0.32% showed three roots, which matches with Cleghorn and Bürklein et al. studies [2, 11]. 6.50% samples had two canals, approximate ranges with other studies. [10, 11, 19]. Only 0.64% showed three canals, similar to Bürklein and Zillich studies [2, 10]. The C-shaped root canal system was represented 7%, different with 0.7% in India [17] 0.9–2% in the Iranian population [1, 4], and 0.6% in China [19]. The highest C-shape root canal system according to Fan criteria [22] was C4a, which could not be correlated with any other study. The most frequent canal bifurcation was in the middle third, similar with Liu and Jaju et al. results [9, 31]. Radicular grooves were located in the mesial area nearly to the lingual zone, similar to other findings [1, 9, 20–22, 25, 27].

This study reported four cases of mandibular second premolars that could not be classified according to Fan criteria. Twos cases with a single root and single canal, two with a single root and two canals; Canal bifurcation level was located in one case in the cervical third and three cases in the middle third. Radicular grooves were located in the buccal area in three samples, and the lingual area in another sample.

According to the gender and C-shaped configuration to Fan criteria to the 100% of the C-shaped samples studied, male patients showed 47.72% and Female patients showed 52.27%. Statistical analyses showed that women had a slightly more incidence of C-shape mandibular premolars, similar to Huang and Sert findings [32, 33]. However most other investigations did not pay a special attention to compare the difference between male and female patients studied [34].

The importance of a proper diagnosis and analysis of cases with anatomical variations implies the application of rigorous criteria in clinical action. In cases of mandibular premolars with C-shaped root canal system configurations, use of magnification and instrumentation with anti-curvature technique is recommended due to the roots concavities, dentinal thinness walls on the lingual zone near to the mesial area and their narrow canals. Excessive dentinal remove could cause band perforations, transportation, or apical perforation. It is necessary the use of copious irrigation with sodium hypochlorite, ultrasonic activation to clean the presence of isthmus and use of EDTA. Obturation with thermoplastic gutta-percha technique is recommended, thus guarantees the success of the therapy or reduces its failure [6, 35].

## Conclusions

Mandibular premolars in the Venezuelan population exhibited great variability in root canal morphology, for this reason, clinicians should be aware of the anatomical variations of these teeth and should evaluate each case carefully, clinically and radiographically. In case of any doubt, CBCT should be indicated for treatment planning. This tool will show all details and variations present, and lead to successful endodontic treatment.

## Data Availability

The datasets used and/or analyzed during the current study are available from the corresponding author on reasonable request..
